# Supplementary material for: T cell migration requires ion and water influx to regulate actin polymerization
Source: Nat Commun. 2023 Dec 6;14:7844. doi: 10.1038/s41467-023-43423-8 (PMC10700356; doi:10.1038/s41467-023-43423-8)
Supplement: Supplementary file 1 — Supplementary Information [file 41467_2023_43423_MOESM1_ESM.pdf]

T cell migration requires ion and water influx to regulate actin polymerization

Supplementary Information

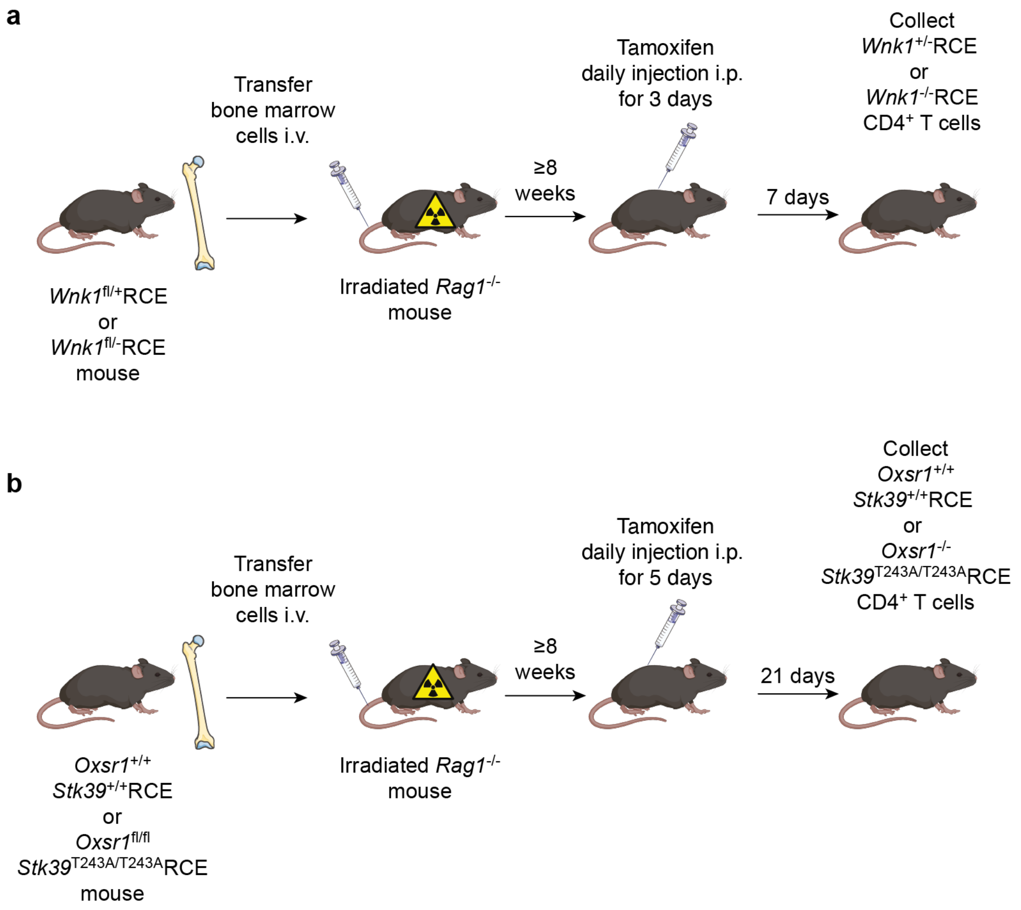

Supplementary Figure 1. Generation of T cells deficient in WNK1 or deficient in OXSR1 with a mutation in STK39.

(**a**) Bone marrow from *Wnk1*$^{fl/+}$RCE or *Wnk1*$^{fl/fl}$RCE mice was transferred i.v. into irradiated RAG1-deficient (*Rag1*$^{-/-}$) mice. At least 8 weeks later, mice were treated with three daily injections of tamoxifen i.p. and CD4^{+} T cells purified from lymph nodes 7 d after the start of tamoxifen injection. (**b**) Bone marrow from *Oxsr1*$^{+/+}$*Stk39*$^{+/+}$RCE or *Oxsr1*$^{fl/fl}$*Stk39*$^{T243A/T243A}$RCE mice was transferred i.v. into irradiated RAG1-deficient (*Rag1*$^{-/-}$) mice. At least 8 weeks later, mice were treated with five daily injections of tamoxifen i.p. and CD4^{+} T cells purified from lymph nodes 21 d after the start of tamoxifen injection. RCE, *ROSA26*^{CreERT2}. Mouse image from Biorender.

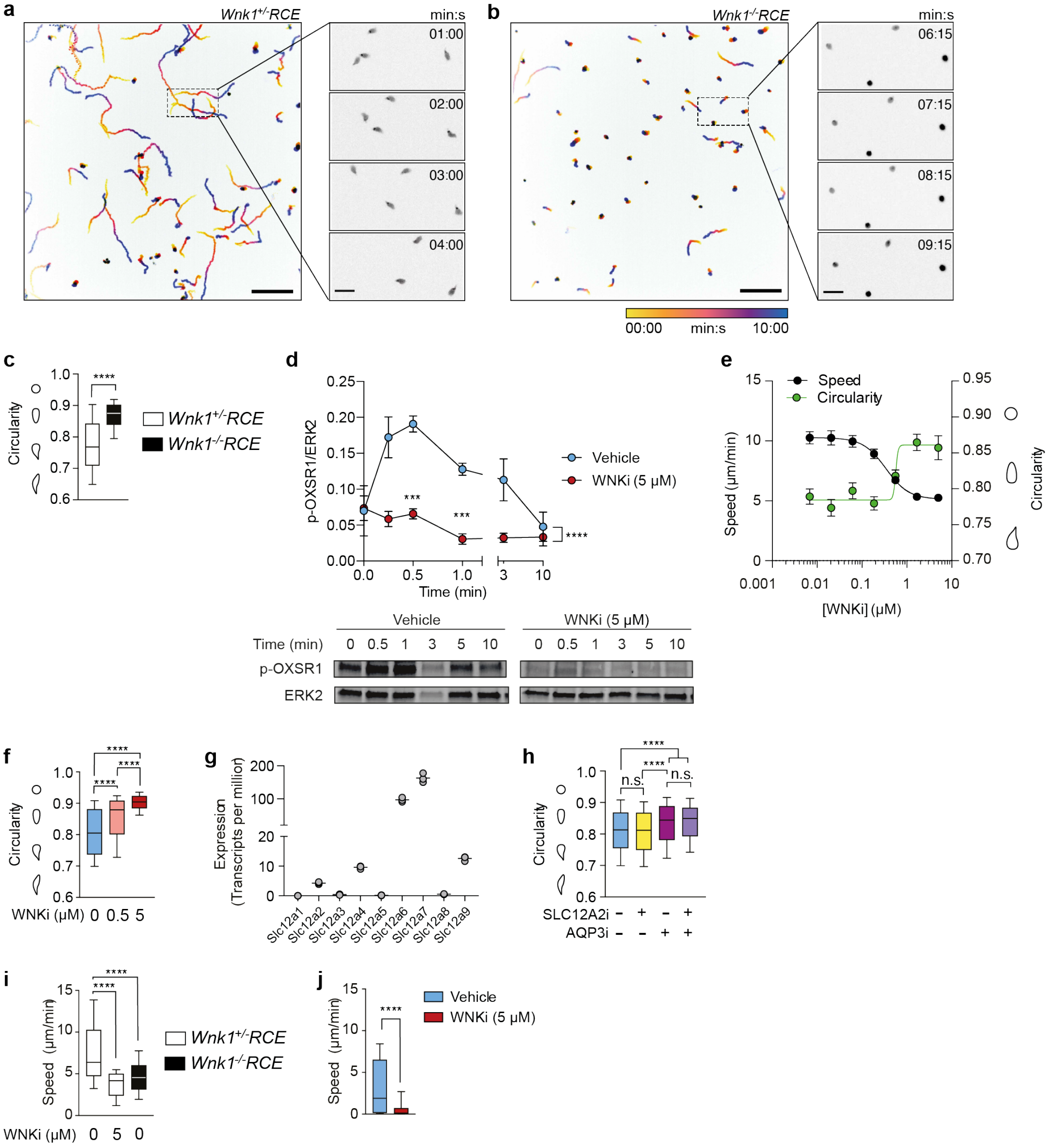

Supplementary Figure 2. WNK1 pathway proteins are required for CCL21-induced migration and polarization of naive and activated CD4⁺ T cells.

(**a-b**) Time-projected example images of CellTrace Violet-labelled mouse naïve CD4⁺ T cells of the indicated genotypes migrating on ICAM-1 under agarose in response to CCL21. Scale bar: 100 μm, zoomed section scale bar: 20 μm. (**c**) Circularity (example cell shapes on Y-axis) of mouse naïve CD4⁺ T cells migrating under agarose in response to

CCL21 of the indicated genotypes. (**d**) Mean (±SEM) levels of phosphorylated OXSR1 (p-OXSR1), normalized to the levels of ERK2, in mouse naïve CD4$^+$ T cells stimulated with CCL21 for the indicated times, determined by immunoblotting total cell lysates with antibodies to p-OXSR1 and ERK2. Example immunoblots shown below graph. (**e**) Mean (±95% CI) speed and circularity (example shapes on y-axis) of mouse naive CD4$^+$ T cells treated with different concentrations of WNKi, migrating on ICAM-1 under agarose in response to CCL21. (**f**) Circularity (example cell shapes on Y-axis) of mouse naïve CD4$^+$ T cells migrating under agarose in response to CCL21 in the presence of different doses of WNK1 inhibitor. (**g**) mRNA levels of genes encoding SLC12A-family ion co-transporters in mouse naïve CD4 T cells from RNA-seq analysis[6]. Systematic names with alternative common names in parentheses: SLC12A1 (NKCC2), SLC12A2 (NKCC1), SLC12A3 (NCC), SLC12A4 (KCC1), SLC12A5 (KCC2), SLC12A6 (KCC3), SLC12A7 (KCC4), SLC12A8 (CCC9), SLC12A9 (CIP1). Line indicates mean. (**h**) Circularity (example cell shapes on Y-axis) of mouse naïve CD4$^+$ T cells migrating under agarose in response to CCL21 in the presence of the indicated inhibitors. (**i**) Speed of mouse activated CD4$^+$ T cells migrating under agarose in ICAM-1 coated dishes in response to CXCL12. (**j**) Speed of mouse activated CD4$^+$ T cells migrating directionally in a collagen-I matrix in response to a CXCL12 chemokine gradient, with or without WNKi. WNKi, WNK inhibitor (WNK463); SLC12A2i, SLC12A2 inhibitor (bumetanide); AQP3, AQP3 inhibitor (DFP00173). Sample numbers: *Wnk1*$^{+/-}$RCE, *n* = 322 cells; *Wnk1*$^{-/-}$RCE, *n* = 386 cells (c); *n* = 3 mice (d, g); WNKi 0.007 μM, speed: *n* = 532 cells, circularity: *n* = 365 cells; WNKi 0.02 μM, speed: *n* = 505 cells, circularity: *n* = 282 cells; WNKi 0.06 μM, speed: *n* = 527 cells, circularity: *n* = 232 cells; WNKi 0.19 μM, speed: *n* = 570 cells, circularity: *n* = 354 cells; WNKi 0.6 μM, speed: *n* = 447 cells, circularity: *n* = 274 cells; WNKi 1.7 μM, speed: *n* = 422 cells, circularity: *n* = 134 cells; WNKi 5 μM, speed: *n* = 522 cells, circularity: *n* = 122 cells (e); Vehicle, *n* = 365 cells; 0.5 μM WNKi, *n* = 274 cells; 5 μM WNKi, *n* = 122 cell (f); Vehicle,

n = 1791 cells; SLC12A2i, *n* = 1978 cells; AQP3i, *n* = 1754 cells; SLC12A2i + AQP3i, *n* = 1720 cells (h); *Wnk1*$^{+/-}$RCE, *n* = 842 cells; *Wnk1*$^{+/-}$RCE + WNKi, *n* = 829 cells; *Wnk1*$^{-/-}$ RCE, *n* = 806 cells (i); Vehicle, *n* = 43 cells; WNKi, *n* = 42 cells (j). Data are pooled from 2 (c, i), 3 (d), 4 (h) or 1 (g, j) independent experiment(s), or from one experiment representative of 2 independent experiments (e, f). Box-plot centre line, median; box limits, 75th and lower 25th percentiles; whiskers, 10th and 90th percentiles. Statistical analysis was carried using 2-way ANOVA (d), 2-sided Mann-Whitney (c) or Kruskal-Wallis test (f, h-i); n.s., not significant, ****p or q< 0.0001. Source data are provided as a Source Data file.

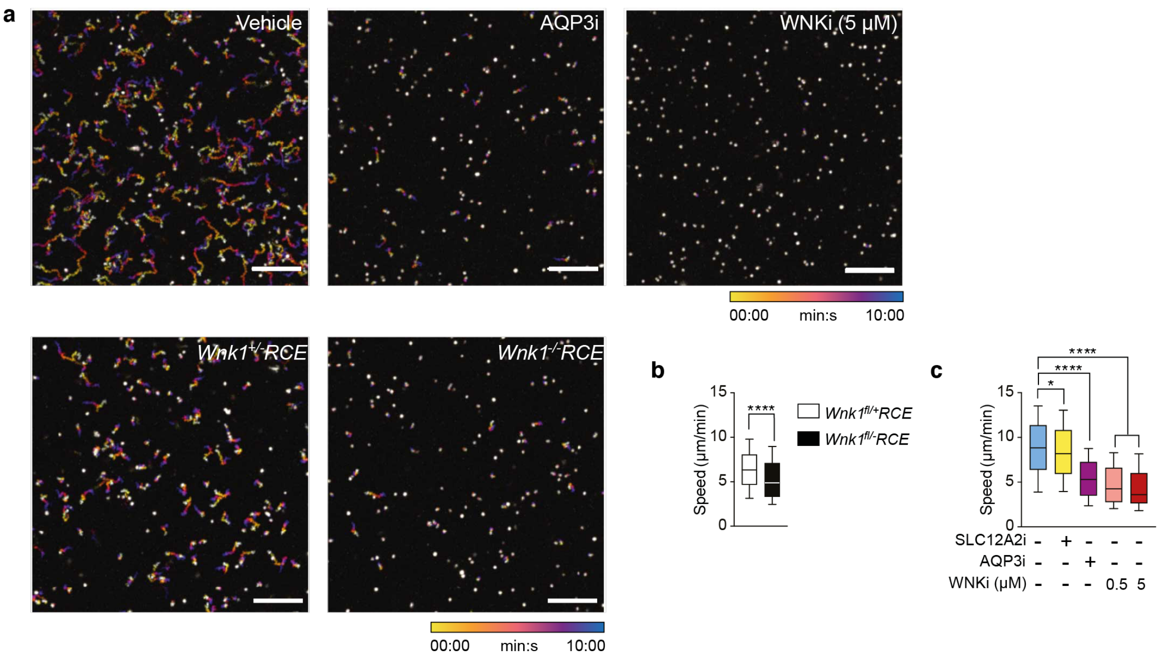

Supplementary Figure 3. The WNK1 pathway controls T cell migration in a 3D collagen matrix.

(**a**) Z- and time-projected example images of CellTrace Violet-labelled mouse naïve CD4$^+$ T cells of the indicated genotypes, or treated with the indicated inhibitors migrating in a collagen-I matrix in response to CCL21. Scale bar: 100 µm. (**b, c**) Speed of mouse naive CD4$^+$ T cells of the indicated genotypes or treated with inhibitors migrating in a collagen-I matrix in response to CCL21. WNKi, WNK inhibitor (WNK463); SLC12A2i, SLC12A2 inhibitor (bumetanide); AQP3, AQP3 inhibitor (DFP00173). Sample numbers: *Wnk1*$^{+/-}$RCE, *n* = 188 cells; *Wnk1*$^{-/-}$RCE, *n* = 310 cells (b). Vehicle, *n* = 637 cells; SLC12A2i, *n* = 649 cells; AQP3i, *n* = 619 cells; 0.5 µM WNKi, *n* = 640 cells; 5 µM WNKi, *n* = 491 cells (c). Data are from 1 experiment (b) or pooled from 2 experiments (c). Box-plot centre line, median; box limits, 75th and lower 25th percentiles; whiskers, 10th and 90th percentiles. Statistical analysis was carried using a 2-sided Mann-Whitney (b) or Kruskal-Wallis test (c); n.s., not significant, *0.01 < *q* < 0.05, ****p or q < 0.0001. Source data are provided as a Source Data file.

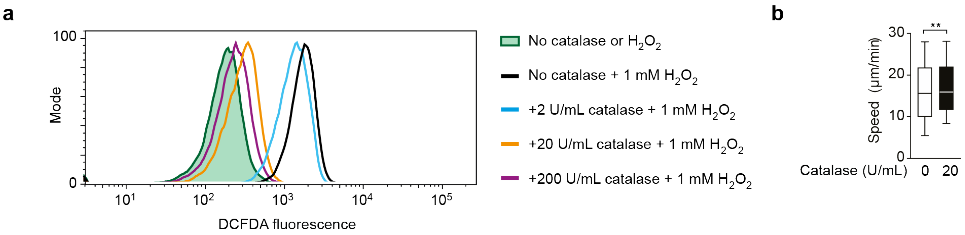

Supplementary Figure 4. Extracellular H_2O_2 is not required for T cell migration under agarose.

(**a**) Representative flow cytometry plot of mouse naïve $CD4^+$ T cells stained with the reactive oxygen species indicator DCFDA, pre-incubated with catalase at the indicated concentrations and gated on live cells. Cells were treated with 1 mM H_2O_2 for 30 min and analyzed using flow cytometry. (**b**) Speed of mouse naïve $CD4^+$ T cells pre-treated with or without 20 U/mL catalase, migrating on ICAM-1 under agarose with or without 20 U/mL catalase. Plot representative of 2 experiments (a). Sample numbers: no catalase, $n = 1447$ cells; 20 U/mL catalase, 2081 cells (b). Data are pooled from 2 experiments. Box-plot centre line, median; box limits, 75th and lower 25th percentiles; whiskers, 10th and 90th percentiles. Statistical analysis was carried using a 2-sided Mann-Whitney test. **0.01 < p < 0.001. Source data are provided as a Source Data file.

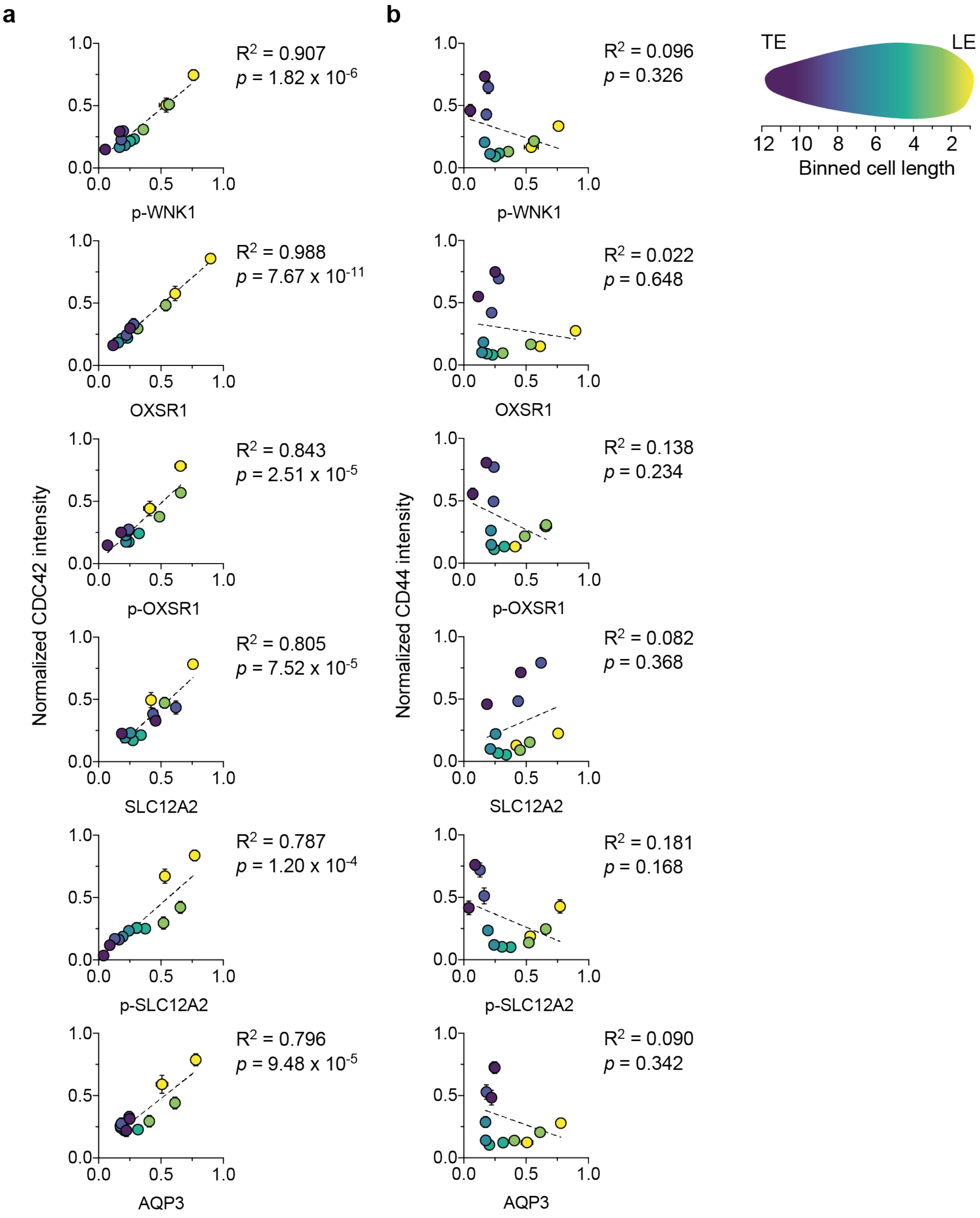

Supplementary Figure 5. WNK1 pathway proteins polarize to the leading edge of migrating CD4⁺ T cells.

(**a, b**) Mouse naïve CD4⁺ T cells migrating in response to CCL21 on ICAM-1 were fixed and stained with antibodies against the indicated proteins. Graphs show normalized fluorescence intensity of p-WNK1, OXSR1, p-OXSR1, SLC12A2, p-SLC12A2 and AQP3 versus the normalized fluorescence intensity of CDC42 (a) or CD44 (b), markers of the

leading and trailing edges of the cell, respectively. Dots represent the mean (±SEM) fluorescence intensity, at different positions along the length of the cell as indicated by the colors representing 12 bins from the leading to the trailing edge (see legend). This is the same data that is shown in Figure 3h. LE, leading edge; TE, trailing edge. Sample numbers: p-WNK1, *n* = 47 cells; OXSR1, *n* = 68 cells; p-OXSR1, *n* = 59 cells; SLC12A2, *n* = 58 cells; p-SLC12A2, *n* = 62 cells; AQP3, *n* = 62 cells. Data are pooled from 2 (OXSR1, SLC12A2) or 3 (p-WNK1, p-OXSR1, p-SLC12A2, AQP3) experiments. *p*-values determined using Pearson's correlation test; R, correlation coefficient. Source data are provided as a Source Data file.

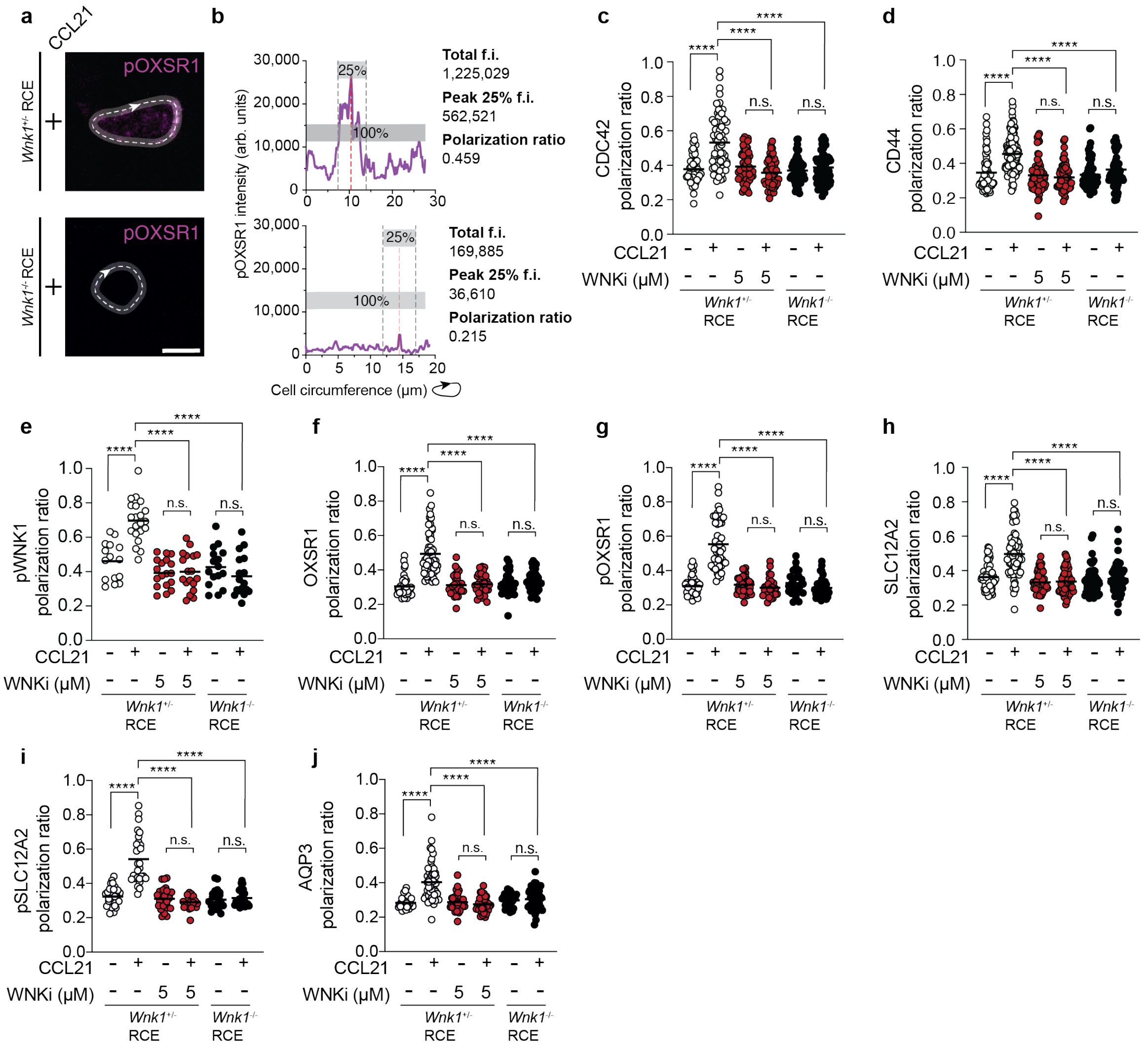

Supplementary Figure 6. WNK1-dependent polarization of WNK1 pathway proteins and their activities in migrating T cells.

Mouse naïve CD4 T cells of the indicated genotypes migrating in response to CCL21, with or without WNK463 in ICAM-1 coated dishes, were fixed, stained for the indicated proteins, and imaged by confocal microscopy. (**a**) Example images of cells stained for p-OXSR1 demonstrating how the polarization measure is established. White arrow indicates starting point of line around the circumference of the cell. Scale bar, 5 μm. (**b**) Intensity profile of p-OXSR1 along the circumference as measured in a; arb. units, arbitrary units. To calculate the polarization ratio, the sum of 25% of the fluorescence intensity (f.i.) signal

(grey dashed lines) centered around the peak intensity point (red dashed line) is divided by the sum of the f.i. of the entire circumference. (**c-j**) Graphs showing the polarization ratio for the indicated proteins. Each dot represents the polarization ratio of a single cell; lines represent mean. WNKi, WNK inhibitor (WNK463). Sample numbers: Unstimulated *Wnk1*$^{+/-}$RCE, *n* = 48 cells; Stimulated *Wnk1*$^{+/-}$RCE, *n* = 72 cells; Unstimulated WNKi - treated *Wnk1*$^{+/-}$RCE, *n* = 54 cells; Stimulated WNKi-treated *Wnk1*$^{+/-}$RCE, *n* = 50 cells; Unstimulated *Wnk1*$^{-/-}$RCE, *n* = 55 cells; Stimulated *Wnk1*$^{-/-}$RCE, *n* = 72 cells (c); Unstimulated *Wnk1*$^{+/-}$RCE, *n* = 56 cells; Stimulated *Wnk1*$^{+/-}$RCE, *n* = 123 cells; Unstimulated WNKi-treated *Wnk1*$^{+/-}$RCE, *n* = 68 cells; Stimulated WNKi-treated *Wnk1*$^{+/-}$RCE, *n* = 65 cells; Unstimulated *Wnk1*$^{-/-}$RCE, *n* = 65 cells; Stimulated *Wnk1*$^{-/-}$RCE, *n* = 63 cells (d); Unstimulated *Wnk1*$^{+/-}$RCE, *n* = 15 cells; Stimulated *Wnk1*$^{+/-}$RCE, *n* = 21 cells; Unstimulated WNKi-treated *Wnk1*$^{+/-}$RCE, *n* = 18 cells; Stimulated WNKi-treated *Wnk1*$^{+/-}$RCE, *n* = 18 cells; Unstimulated *Wnk1*$^{-/-}$RCE, *n* = 16 cells; Stimulated *Wnk1*$^{-/-}$RCE, *n* = 18 cells (e); Unstimulated *Wnk1*$^{+/-}$RCE, *n* = 35 cells; Stimulated *Wnk1*$^{+/-}$RCE, *n* = 52 cells; Unstimulated WNKi-treated *Wnk1*$^{+/-}$RCE, *n* = 29 cells; Stimulated WNKi-treated *Wnk1*$^{+/-}$RCE, *n* = 30 cells; Unstimulated *Wnk1*$^{-/-}$RCE, *n* = 32 cells; Stimulated *Wnk1*$^{-/-}$RCE, *n* = 38 cells (f); Unstimulated *Wnk1*$^{+/-}$RCE, *n* = 40 cells; Stimulated *Wnk1*$^{+/-}$RCE, *n* = 46 cells; Unstimulated WNKi-treated *Wnk1*$^{+/-}$RCE, *n* = 26 cells; Stimulated WNKi-treated *Wnk1*$^{+/-}$RCE, *n* = 36 cells; Unstimulated *Wnk1*$^{-/-}$RCE, *n* = 40 cells; Stimulated *Wnk1*$^{-/-}$RCE, *n* = 26 cells (g); Unstimulated *Wnk1*$^{+/-}$RCE, *n* = 59 cells; Stimulated *Wnk1*$^{+/-}$RCE, *n* = 76 cells; Unstimulated WNKi-treated *Wnk1*$^{+/-}$RCE, *n* = 58 cells; Stimulated WNKi-treated *Wnk1*$^{+/-}$RCE, *n* = 59 cells; Unstimulated *Wnk1*$^{-/-}$RCE, *n* = 58 cells; Stimulated *Wnk1*$^{-/-}$RCE, *n* = 52 cells (h); Unstimulated *Wnk1*$^{+/-}$RCE, *n* = 29 cells; Stimulated *Wnk1*$^{+/-}$RCE, *n* = 28 cells; Unstimulated WNKi-treated *Wnk1*$^{+/-}$RCE, *n* = 22 cells; Stimulated WNKi-treated *Wnk1*$^{+/-}$RCE, *n* = 25 cells; Unstimulated *Wnk1*$^{-/-}$RCE, *n* = 26 cells; Stimulated *Wnk1*$^{-/-}$RCE, *n* = 29 cells (i); Unstimulated *Wnk1*$^{+/-}$RCE, *n* = 36 cells; Stimulated *Wnk1*$^{+/-}$RCE, *n* = 53 cells;

Unstimulated WNKi-treated *Wnk1*$^{+/-}$RCE, n = 42 cells; Stimulated WNKi-treated *Wnk1*$^{+/-}$RCE, n = 35 cells; Unstimulated *Wnk1*$^{-/-}$RCE, n = 33 cells; Stimulated *Wnk1*$^{-/-}$RCE, n = 39 cells (j). Data are from 1 (e), or pooled from 2 (f, g, i, j) or 3 (c, d, h) experiments. Statistical analysis using Kruskal-Wallis test with FDR multiple comparisons correction, ****q < 0.0001. n.s., not significant. Source data are provided as a Source Data file.

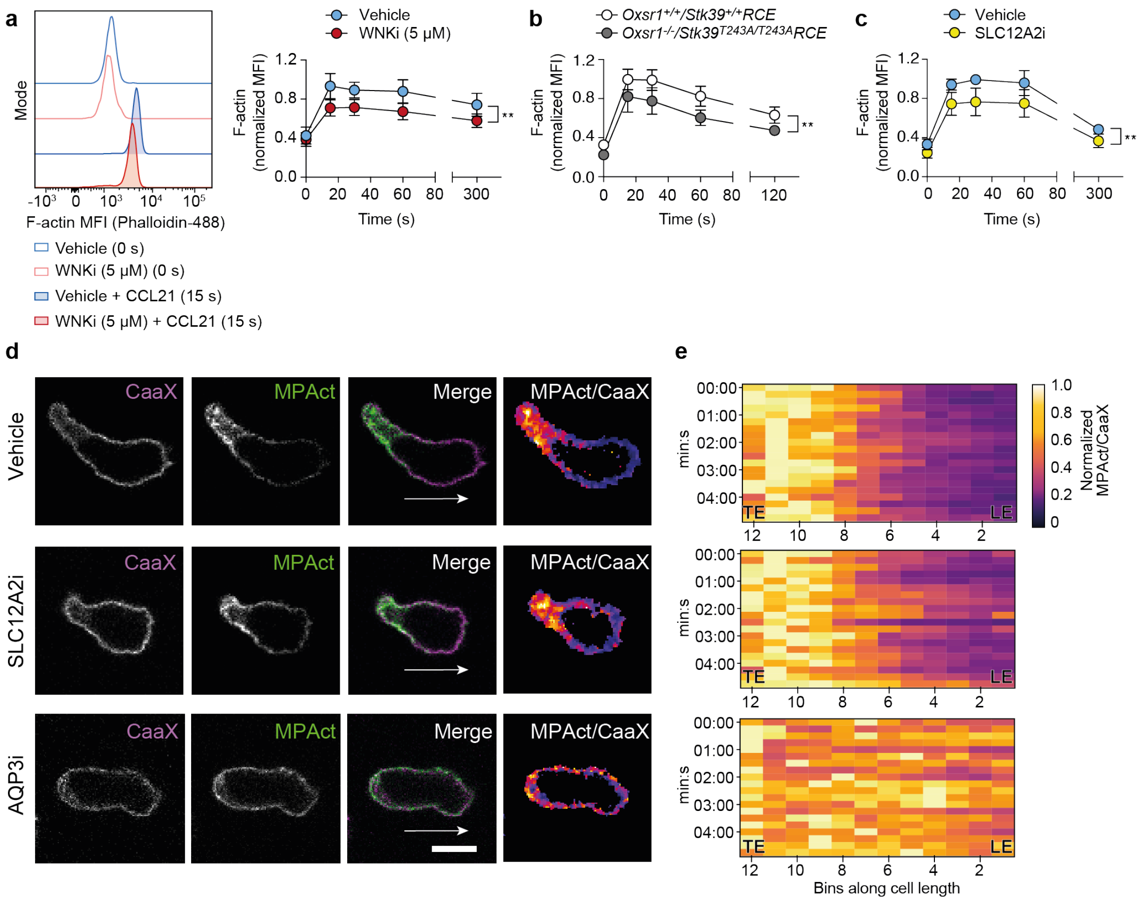

Supplementary Figure 7. The WNK1 pathway regulates F-actin in migrating CD4$^+$ T cells.

(**a-c**) Naïve CD4$^+$ T cells of the indicated genotypes or treated with inhibitor or vehicle, were stimulated with CCL21 for the indicated times and F-actin measured by flow cytometric analysis of phalloidin staining. Example histograms of F-actin mean fluorescence intensity (MFI) of mouse naïve CD4$^+$ T cells treated with or without WNKi, stimulated with CCL21 for the indicated times, measured by flow cytometry (a, left). Graphs show mean (±95% CI) F-actin fluorescence (a, right, b, c). (**d, e**) Mouse naïve CD4$^+$ T cells co-expressing GFP-CaaX (CaaX) and MPAct-mCherry (MPAct), were imaged migrating in response to CCL21 on ICAM-1 under agarose in the presence of inhibitors or vehicle. Cells were imaged every 15 s, for 2.5 or 5 min. Representative images of CaaX and MPAct fluorescence and MPAct/CaaX ratio in cells treated with vehicle or

inhibitors showing direction of migration (white arrow); scale bar, 5 μm (d). Heatmaps showing normalized MPAct/CaaX ratios over time of the three cells shown in D (e). WNKi, WNK inhibitor (WNK463); SLC12A2i, SLC12A2 inhibitor (bumetanide). LE, leading edge; TE, trailing edge. Sample numbers: n = 9 per timepoint and condition (a); n = 10 per timepoint and genotype (b); n = 5 for vehicle 60 s, n = 6 for all other timepoints and conditions (c). Data are pooled from 3 experiments (a-c). Statistical analysis was carried out using 2-way ANOVA (a-b) or a mixed-effects model (c) with FDR correction; **0.001 < q < 0.01. Source data are provided as a Source Data file.

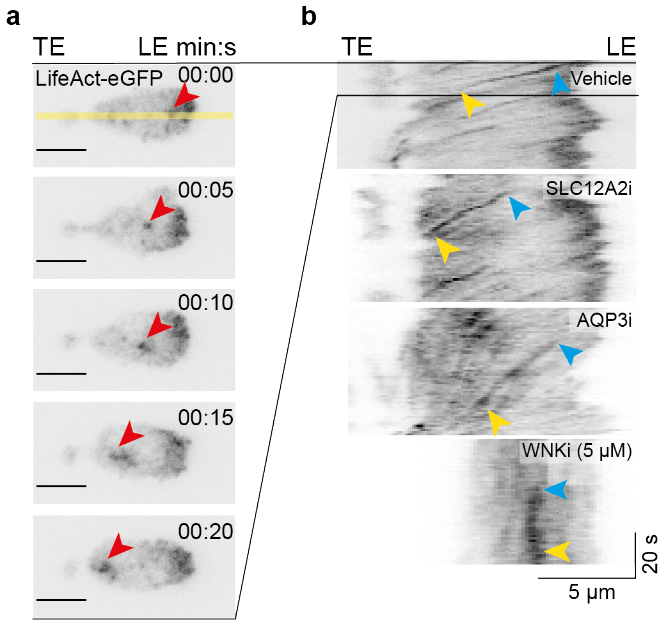

Supplementary Figure 8. Measuring retrograde actin flow in migrating CD4$^+$ T cells. (**a, b**) Total internal reflection fluorescence microscopy of mouse naive CD4$^+$ T cells expressing LifeAct-eGFP migrating on PEG-coated glass under agarose in response to CCL21, treated with inhibitors or vehicle only. LifeAct-eGFP fluorescence was imaged every 1 s for 1 min. Images of vehicle-treated cells at a series of timepoints showing retrograde movement of F-actin (red arrowhead); scale bars, 5 µm (a). Yellow line indicates location of line used for kymograph generation. Representative kymographs of mouse naïve CD4$^+$ T cells treated with vehicle or inhibitors derived from time-lapse imaging as shown in a, taking fluorescence along the length of the cell (b). The top kymograph is taken from the cell in a, blue and yellow arrowheads indicate the start and end of F-actin flow respectively, the slope of the time-dependent F-actin flow was used to calculate the retrograde actin flow rates shown in Figure 6a, b, e. WNKi, WNK inhibitor (WNK463); SLC12A2i, SLC12A2 inhibitor (bumetanide); AQP3, AQP3 inhibitor (DFP00173).

Supplementary Movies

Supplementary Movie 1. CD4$^+$ T cell migration under agarose.

Widefield microscopy of CellTrace Violet (CTV)-labelled *Wnk1*$^{+/-}$RCE or *Wnk1*$^{-/-}$RCE naïve CD4$^+$ T cells, stimulated with CCL21, migrating under agarose on an ICAM-1 coated dish for 10 min, imaged every 15s. Purple, CTV labelled cells; yellow, propidium iodide for dead cell exclusion. Time stamp: min:s. Scale bar, 200 μm.

Supplementary Movie 2. CD4$^+$ T cell migration in a collagen-I matrix.

Z-projected confocal microscopy of CellTrace Violet (CTV)-labelled mouse naïve CD4 T cells from C57BL/6J mice suspended in a collagen-I matrix, stimulated with CCL21 in the presence of the indicated inhibitors, or vehicle only. Cells were imaged every 15 s for 10 min, in a 120 μm z-stack. Black, CTV labelled cells; red, propidium iodide for dead cell exclusion. Time stamp: min:s. Scale bar, 100 μm.

Supplementary Movie 3. GFP-tagged WNK1 pathway proteins in migrating CD4$^+$ T cells.

Confocal microscopy of CD4$^+$ T cells expressing GFP-WNK1, GFP-OXSR1, GFP-SLC21A2 or GFP only migrating on ICAM-1 under agarose in response to CCL21. Cells were imaged every 15 s for 2.5 min. Look-up table GFP intensity: yellow, high; blue-black, low. Time stamp: min:s. Scale bar, 5 μm.

Supplementary Movie 4. Plasma membrane-F-actin spacing in migrating CD4$^+$ T cells.

iSIM imaging of mouse naïve CD4$^+$ T cells expressing LifeAct-eGFP (cyan) migrating on ICAM-1 under agarose in response to CCL21, treated with inhibitors or vehicle only. To

visualize the plasma membrane, cells were labelled with CellMask Orange plasma membrane stain (magenta). Images were taken every 250 ms. Time stamp: s:ms. Scale bar, 5 µm.

Supplementary Movie 5. Membrane-proximal F-actin in migrating CD4$^+$ T cells.

Confocal microscopy of naive CD4$^+$ T cells expressing MPAct-mCherry and GFP-CaaX migrating on ICAM-1 under agarose in response to CCL21, treated with inhibitors or vehicle only. Fluorescence was imaged every 15 s for 2.5 min. Look-up table of MPAct/CaaX ratio intensity: yellow, high; blue-black, low (as in Figure 6a). Time stamp: min:s. Scale bar, 5 µm.

Supplementary Movie 6. Actin retrograde flow in migrating CD4$^+$ T cells.

CD4$^+$ T cells expressing LifeAct-eGFP migrating on PEG-coated glass under agarose in response to CCL21, treated with inhibitors or vehicle only, imaged by TIRF microscopy. LifeAct-eGFP fluorescence was imaged every 1 s for 1 min. Black, LifeAct-eGFP. Time stamp: s. Scale bar, 5 µm.
