# Supplementary material for: T cell migration requires ion and water influx to regulate actin polymerization
Source: Nat Commun. 2023 Dec 6;14:7844. doi: 10.1038/s41467-023-43423-8 (PMC10700356; doi:10.1038/s41467-023-43423-8)
Supplement: Supplementary file 3 — Description of Additional Supplementary Files [file 41467_2023_43423_MOESM3_ESM.pdf]

Supplementary Movies

Supplementary Movie 1. CD4$^+$ T cell migration under agarose.

Widefield microscopy of CellTrace Violet (CTV)-labelled *Wnk1*$^{+/-}$RCE or *Wnk1*$^{-/-}$RCE naïve CD4$^+$ T cells, stimulated with CCL21, migrating under agarose on an ICAM-1 coated dish for 10 min, imaged every 15s. Purple, CTV labelled cells; yellow, propidium iodide for dead cell exclusion. Time stamp: min:s. Scale bar, 200 µm.

Supplementary Movie 2. CD4$^+$ T cell migration in a collagen-I matrix.

Z-projected confocal microscopy of CellTrace Violet (CTV)-labelled mouse naïve CD4 T cells from C57BL/6J mice suspended in a collagen-I matrix, stimulated with CCL21 in the presence of the indicated inhibitors, or vehicle only. Cells were imaged every 15 s for 10 min, in a 120 µm z-stack. Black, CTV labelled cells; red, propidium iodide for dead cell exclusion. Time stamp: min:s. Scale bar, 100 µm.

Supplementary Movie 3. GFP-tagged WNK1 pathway proteins in migrating CD4$^+$ T cells.

Confocal microscopy of CD4$^+$ T cells expressing GFP-WNK1, GFP-OXSR1, GFP-SLC21A2 or GFP only migrating on ICAM-1 under agarose in response to CCL21. Cells were imaged every 15 s for 2.5 min. Look-up table GFP intensity: yellow, high; blue-black, low. Time stamp: min:s. Scale bar, 5 µm.

Supplementary Movie 4. Plasma membrane-F-actin spacing in migrating CD4$^+$ T cells.

iSIM imaging of mouse naïve CD4$^+$ T cells expressing LifeAct-eGFP (cyan) migrating on ICAM-1 under agarose in response to CCL21, treated with inhibitors or vehicle only. To

visualize the plasma membrane, cells were labelled with CellMask Orange plasma membrane stain (magenta). Images were taken every 250 ms. Time stamp: s:ms. Scale bar, 5 µm.

Supplementary Movie 5. Membrane-proximal F-actin in migrating CD4$^+$ T cells.
Confocal microscopy of naive CD4$^+$ T cells expressing MPAct-mCherry and GFP-CaaX migrating on ICAM-1 under agarose in response to CCL21, treated with inhibitors or vehicle only. Fluorescence was imaged every 15 s for 2.5 min. Look-up table of MPAct/CaaX ratio intensity: yellow, high; blue-black, low (as in Figure 6a). Time stamp: min:s. Scale bar, 5 µm.

Supplementary Movie 6. Actin retrograde flow in migrating CD4$^+$ T cells.
CD4$^+$ T cells expressing LifeAct-eGFP migrating on PEG-coated glass under agarose in response to CCL21, treated with inhibitors or vehicle only, imaged by TIRF microscopy. LifeAct-eGFP fluorescence was imaged every 1 s for 1 min. Black, LifeAct-eGFP. Time stamp: s. Scale bar, 5 µm.
