# Supplementary material for: T cell migration requires ion and water influx to regulate actin polymerization
Source: Nat Commun. 2023 Dec 6;14:7844. doi: 10.1038/s41467-023-43423-8 (PMC10700356; doi:10.1038/s41467-023-43423-8)
Supplement: Supplementary file 10 — Reporting Summary [file 41467_2023_43423_MOESM10_ESM.pdf]

nature portfolio

Corresponding author(s): VLJ Tybulewicz

Last updated by author(s): Oct 12, 2023

Reporting Summary

Nature Portfolio wishes to improve the reproducibility of the work that we publish. This form provides structure for consistency and transparency in reporting. For further information on Nature Portfolio policies, see our Editorial Policies and the Editorial Policy Checklist.

Statistics

For all statistical analyses, confirm that the following items are present in the figure legend, table legend, main text, or Methods section.

n/a	Confirmed	
☐	☒	The exact sample size (*n*) for each experimental group/condition, given as a discrete number and unit of measurement
☐	☒	A statement on whether measurements were taken from distinct samples or whether the same sample was measured repeatedly
☐	☒	The statistical test(s) used AND whether they are one- or two-sided<br>*Only common tests should be described solely by name; describe more complex techniques in the Methods section.*
☐	☒	A description of all covariates tested
☐	☒	A description of any assumptions or corrections, such as tests of normality and adjustment for multiple comparisons
☐	☒	A full description of the statistical parameters including central tendency (e.g. means) or other basic estimates (e.g. regression coefficient) AND variation (e.g. standard deviation) or associated estimates of uncertainty (e.g. confidence intervals)
☐	☒	For null hypothesis testing, the test statistic (e.g. *F*, *t*, *r*) with confidence intervals, effect sizes, degrees of freedom and *P* value noted<br>*Give P values as exact values whenever suitable.*
☒	☐	For Bayesian analysis, information on the choice of priors and Markov chain Monte Carlo settings
☒	☐	For hierarchical and complex designs, identification of the appropriate level for tests and full reporting of outcomes
☐	☒	Estimates of effect sizes (e.g. Cohen's *d*, Pearson's *r*), indicating how they were calculated

Our web collection on statistics for biologists contains articles on many of the points above.

Software and code

Policy information about availability of computer code

Data collection	Imaging data was collected using µManager, Zeiss Zen Black, Leica LASX or Olympus CellSens Dimension software. Flow cytometry data was collected using BD FACSDiva. Western blot data was collected using Image Studio.
Data analysis	FIJI (FIJI is just imagej, ImageJ2) (Trackmate, Microvolution and VirusTracker plugins) and Huygens Essential 20 were used for image analysis. Flow cytometry was analyzed using FlowJo version 9. NMR data was analysed using Bruker TopSpin 3.6. Statistical analysis and data visualization were performed using GraphPad Prism versions 8-10 and Adobe Illustrator versions 2020-2023. Microsoft Excel for Mac versions 14-16 were used for data organization.

For manuscripts utilizing custom algorithms or software that are central to the research but not yet described in published literature, software must be made available to editors and reviewers. We strongly encourage code deposition in a community repository (e.g. GitHub). See the Nature Portfolio guidelines for submitting code & software for further information.

Data

Policy information about availability of data

All manuscripts must include a data availability statement. This statement should provide the following information, where applicable:

- Accession codes, unique identifiers, or web links for publicly available datasets
- A description of any restrictions on data availability
- For clinical datasets or third party data, please ensure that the statement adheres to our policy

Mouse strains and plasmids available on request. Source data are provided with this paper in a Source Data file. Other data that support the findings of this study

are available from the corresponding author upon reasonable request. RNA sequencing data used in this study are deposited under SRA accession number SRP059425 [https://trace.ncbi.nlm.nih.gov/Traces/?view=study&acc=SRP059425].

Research involving human participants, their data, or biological material

Policy information about studies with human participants or human data. See also policy information about sex, gender (identity/presentation), and sexual orientation and race, ethnicity and racism.

Reporting on sex and gender	Not applicable.
Reporting on race, ethnicity, or other socially relevant groupings	Not applicable.
Population characteristics	Not applicable.
Recruitment	Not applicable.
Ethics oversight	Not applicable.

Note that full information on the approval of the study protocol must also be provided in the manuscript.

Field-specific reporting

Please select the one below that is the best fit for your research. If you are not sure, read the appropriate sections before making your selection.

☒ Life sciences ☐ Behavioural & social sciences ☐ Ecological, evolutionary & environmental sciences

For a reference copy of the document with all sections, see nature.com/documents/nr-reporting-summary-flat.pdf

Life sciences study design

All studies must disclose on these points even when the disclosure is negative.

Sample size	No statistical methods were used to predetermine sample sizes, and sample sizes were chosen empirically. The in vitro experiments consist of multiple cells from multiple mice. The sample sizes were chosen based on analysis of comparable studies performed by others in the field of T cell migration (e.g. Hons et al, Nat Imm 2018).
Data exclusions	For migration experiments, dead cells (as determined by propidium ioidide uptake) were removed. Cells that were in the imaging field for less than half of the video length were also excluded. For protein polarization experiments, only polarized cells (as determined by CDC42 and CD44 staining) were analyzed. These exclusion strategies were pre-determined.
Replication	All experiments were performed multiple times, and all replication attempts were successful. At least two independent experiments with multiple biological replicates per experiment were performed, with similar results for each. Exact replication numbers are in the figure legends of all figures.
Randomization	No randomization was performed for in vitro experiments. For most experiments cells were pooled from multiple mice (except where specifically indicated), and cells from each pool of cells were further divided into different treatment conditions (e.g. ±inhibitor, ±stimulus) to allow for direct comparison between conditions. Thus, no co-variate analysis was required.
Blinding	The investigators were not blinded to allocation during experiments and outcome assessment. Data such as tracking was performed using unbiased image analysis pipelines in FIJI. For experiments with manual analysis, pre-determined exclusion strategies (see above) were applied to avoid bias in the analysis. Analysis pipelines were also designed prior to analysis, and adhered to strictly.

Reporting for specific materials, systems and methods

We require information from authors about some types of materials, experimental systems and methods used in many studies. Here, indicate whether each material, system or method listed is relevant to your study. If you are not sure if a list item applies to your research, read the appropriate section before selecting a response.

Materials & experimental systems

n/a	Involved in the study
☐	☒ Antibodies
☒	☐ Eukaryotic cell lines
☒	☐ Palaeontology and archaeology
☐	☒ Animals and other organisms
☒	☐ Clinical data
☒	☐ Dual use research of concern
☒	☐ Plants

Methods

n/a	Involved in the study
☒	☐ ChIP-seq
☐	☒ Flow cytometry
☒	☐ MRI-based neuroimaging

Antibodies

Antibodies used

Microscopy:
Primary antibodies: anti-CDC42 (B-8 mouse monoclonal, Santa Cruz), biotin-conjugated anti-CD44 (IM7 rat monoclonal, BioLegend), anti-p-WNK1 (S382), anti-OXSR1, anti-p-OXSR1 (S325), anti-SLC12A2, anti-p-SLC12A2 (T203/T207/T212) (all sheep polyclonal sera from MRC Protein Phosphorylation Unit (MRC-PPU)) or anti-AQP3 (NBP1-97927 rabbit polyclonal, Novus Biologicals). All primary antibodies were used at 1:200 dilution.

Secondary antibodies: streptavidin (S11223, Molecular Probes, AF488); goat anti-mouse (A-21424, Invitrogen, AF555), rabbit anti-mouse (A-21427, Invitrogen, AF555), goat anti-rabbit (A21127, Invitrogen, AF647), donkey anti-sheep (A-21245, Invitrogen, AF647). All secondaries were used at 1:250 dilution.

Immunoblotting:
Primary antibodies: anti-OXSR1 (polyclonal, sheep serum, MRC-PPU), anti p-OXSR1 (polyclonal, sheep serum, MRC-PPU), anti-ERK2 (9108, rabbit, Cell Signaling Technologies). All primary antibodies were diluted 1:500.

Secondary antibodies: anti-sheep IgG (H+L) AF680, donkey, Invitrogen; anti-rabbit IgG (H+L) WesternDot 800 goat, Invitrogen. All secondaries were diluted 1:1000.

Validation

anti-CDC42 has been validated by others, and has been cited in 169 publications.
anti-CD44 has been validated by others and cited by 86 publications.
anti-AQP3 has been validated by Western blotting and is cited in 7 publications.
anti-OXSR1 polyclonal antibody has been validated using Western blotting for the correct size. It has been cited in 3 publications.
anti-ERK2 has been validated Western blotting, and cited in 66 publications.

The signalsfrom all phospho-specific antibodies were shown to be dependent on WNK1 activity: using either WNK1-deficient T cells or with WNKi treatment phospho signals became undetectable. All secondary antibodies have been confirmed to bind a target using secondary only validation.

Animals and other research organisms

Policy information about studies involving animals; ARRIVE guidelines recommended for reporting animal research, and Sex and Gender in Research

Laboratory animals

All mice were on a C57BL/6 background. C57BL/6J mice were used as wild-type animals. In addition, mice carrying the following genetically modified alleles were used: Rosa26CreERT2 (RCE), Wnk1fl, Wnk1-/-, Oxsr1fl, Stk39T234A, Slc12a2-/-, LifeAct-GFP+, and Rag1-. For more details on mouse strains see the Methods section.

Mice were kept under controlled 12 h light/dark cycles in individually ventilated cages under specific pathogen-free conditions. Mice were kept at 23ºC and 50% humidity, with unlimited access to food and water. CD4+ T cells were isolated from the lymph nodes of mice that were at least 6 weeks of age. Both sexes were used. In all experiments, mice were age- and sex-matched.

Wild animals

The study did not involve wild animals.

Reporting on sex

Mice were sex-matched within an experiment. Both male and female mice were used; sex-based analysis was not performed.

Field-collected samples

No field-collected samples were used.

Ethics oversight

All experiments were approved by the Francis Crick Institute Animal Welfare Ethical Review Board and were carried out under the authority of a Project Licence granted by the UK Home Office.

Note that full information on the approval of the study protocol must also be provided in the manuscript.

Plants

Seed stocks	Not applicable.
Novel plant genotypes	Not applicable.
Authentication	Not applicable.

Flow Cytometry

Plots

Confirm that:

- [x] The axis labels state the marker and fluorochrome used (e.g. CD4-FITC).
- [x] The axis scales are clearly visible. Include numbers along axes only for bottom left plot of group (a 'group' is an analysis of identical markers).
- [ ] All plots are contour plots with outliers or pseudocolor plots.
- [ ] A numerical value for number of cells or percentage (with statistics) is provided.

Methodology

Sample preparation	Mouse T cells were isolated as described in the Methods section, and rested overnight. Cells were stimulated with CCL21 at the indicated timepoint, fixed with 4% paraformaldehyde and stained with iFluor 488-conjugated phalloidin (Abcam ab176753).<br>For cell sorting of WNK1 pathway GFP-tagged cells, CD4+ T cells were isolated and rested overnight. The next day GFP+ cells were sorted.<br>For cell sorting of MPAct and 8xGFP expressing T cells, CD4+ T cells were isolated and electroporated with MPAct or 8xGFP-expressing plasmids,and rested overnight. The next day, either GFP+ or mCherry and GFP double expressing T cells were sorted.
Instrument	BD LSRII, Avalon
Software	FACSDiva, ProSort
Cell population abundance	For cell sorting experiments, WNK1 pathway GFP-tagged cells were around 2-10% of total isolated T cells. For MPAct experiments, double positive cells represented around 0.5-5% of total isolated T cells.<br><br>Purified naive CD4+ T cells were consistently > 90% pure.
Gating strategy	Lymphocytes were gated from the total sample using FSC/SSC. Because staining and sorting were performed on isolated CD4 T cells, and no specific gating on cell subsets was performed, no example gating plots are included. Cell purity was routinely measured to ensure that cell isolation protocols met the necessary CD4+ T cell purity levels (>90%). Example histograms of fluorescence intensity are shown in Supplementary Figures 4a and 7a.

- [x] Tick this box to confirm that a figure exemplifying the gating strategy is provided in the Supplementary Information.
